# Ginsenoside Rg3 Attenuates TNF-α-Induced Damage in Chondrocytes through Regulating SIRT1-Mediated Anti-Apoptotic and Anti-Inflammatory Mechanisms

**DOI:** 10.3390/antiox10121972

**Published:** 2021-12-10

**Authors:** Ching-Hou Ma, Wan-Ching Chou, Chin-Hsien Wu, I-Ming Jou, Yuan-Kun Tu, Pei-Ling Hsieh, Kun-Ling Tsai

**Affiliations:** 1 Department of Orthopedics, E-Da Hospital, Kaohsiung City 824, Taiwan; ed100771@edah.org.tw (C.-H.M.); T66051040@pt.ncku.edu.tw (W.-C.C.); ed103116@edah.org.tw (C.-H.W.); ed109325@edah.org.tw (I.-M.J.); ed100130@edah.org.tw (Y.-K.T.); 2 School of Medicine for International Students, College of Medicine, I-Shou University, Kaohsiung City 824, Taiwan; 3 Department of Physical Therapy, College of Medicine, National Cheng Kung University, Tainan 701, Taiwan; 4 Department of Anatomy, School of Medicine, China Medical University, Taichung 404, Taiwan; 5 Institute of Allied Health Sciences, College of Medicine, National Cheng Kung University, Tainan 701, Taiwan

**Keywords:** ginsenoside Rg3, chondrocytes, SIRT1, apoptosis, inflammation

## Abstract

The upregulation of tumor necrosis factor-alpha (TNF-α) is a common event in arthritis, and the subsequent signaling cascade that leads to tissue damage has become the research focus. To explore a potential therapeutic strategy to prevent cartilage degradation, we tested the effect of ginsenoside Rg3, a bioactive component of *Panax ginseng*, on TNF-α-stimulated chondrocytes.TC28a2 Human Chondrocytes were treated with TNF-α to induce damage of chondrocytes. SIRT1 and PGC-1a expression levels were investigated by Western blotting assay. Mitochondrial SIRT3 and acetylated Cyclophilin D (CypD) were investigated using mitochondrial isolation. The mitochondrial mass number and mitochondrial DNA copy were studied for mitochondrial biogenesis. MitoSOX and JC-1 were used for the investigation of mitochondrial ROS and membrane potential. Apoptotic markers, pro-inflammatory events were also tested to prove the protective effects of Rg3. We showed Rg3 reversed the TNF-α-inhibited SIRT1 expression. Moreover, the activation of the SIRT1/PGC-1α/SIRT3 pathway by Rg3 suppressed the TNF-α-induced acetylation of CypD, resulting in less mitochondrial dysfunction and accumulation of reactive oxygen species (ROS). Additionally, we demonstrated that the reduction of ROS ameliorated the TNF-α-elicited apoptosis. Furthermore, the Rg3-reverted SIRT1/PGC-1α/SIRT3 activation mediated the repression of p38 MAPK, which downregulated the NF-κB translocation in the TNF-α-treated cells. Our results revealed that administration of Rg3 diminished the production of interleukin 8 (IL-8) and matrix metallopeptidase 9 (MMP-9) in chondrocytes via SIRT1/PGC-1α/SIRT3/p38 MAPK/NF-κB signaling in response to TNF-α stimulation. Taken together, we showed that Rg3 may serve as an adjunct therapy for patients with arthritis.

## 1. Introduction

A variety of cytokines act critical roles, such as tumor necrosis factor (TNF-α), in osteoarthritis (OA) or rheumatoid arthritis (RA) [1]. These pro-inflammatory cytokines have been known to cause cell death [2] and stimulate the synthesis of various inflammatory mediators and cartilage-degrading proteinases that compromise the integrity of cartilage. For instance, an enhancement of interleukin 8 (IL-8) has been observed in OA and RA patients [3], which may induce chondrocyte hypertrophic differentiation [4] and contribute to the development of degenerative joint disorders [5]. Several matrix metalloproteinases (MMPs), such as MMP-1, 3, and 9 have been shown to be produced by OA chondrocytes and implicated in cartilage destruction [6]. Consequently, an approach to suppress the production of these inflammatory mediators or MMPs may be beneficial for patients with these arthropathies.

*Panax ginseng* is a widely used traditional herb in Asia and received considerable attention worldwide due to its medicinal properties. Among the components in ginseng plants, ginsenosides (ginseng saponins) have been known to be the major active ingredients of ginseng and are almost exclusively produced in Panax species. Ginsenosides can be classified into two categories based on the hydroxylation area on the core triterpenoid saponin structure: 20(S)-protopanaxadiol as well as 20(S)-protopanaxatriol. Rg3 is dammarane which is substituted by hydroxy groups at the 3b, 12b as well as 20 pro-S positions, in which the hydroxy group at position 3 has been converted to the reaction of β-D-glucopyranosyl-β-D-glucopyranoside [7]. Besides, several biological activities of ginsenosides have been revealed, such as cardioprotective, anti-cancer, anti-oxidant, or anti-inflammatory effects [7,8]. The majority of the existing research has shown that ginsenoside Rb1 possesses the anti-arthritic effects by various means, such as inhibition of TNF-α upregulation [9], downregulation of reactive oxygen species (ROS), and MMPs production [10], and suppression of mitochondrial permeability transition and apoptosis [11]. Ginsenoside Rg3 also has been revealed to protect chondrocytes by suppressing chondrocyte senescence and the expression of MMP-1 and MMP-13 in the IL-1β-treated chondrocytes [12,13]. Nevertheless, the detailed mechanism underlying the beneficial effects of ginsenoside Rg3 on chondrocytes remains largely unknown.

In the study, we aimed to examine the anti-arthritic property of ginsenoside Rg3 on the TNF-α-stimulated chondrocytes. We analyzed the secretion of IL-8 and the expression of MMP-9 of ginsenoside Rg3-treated cells in response to TNF-α treatment. Furthermore, we investigated the detailed molecular mechanism underlying the pharmacological activities of ginsenoside Rg3. These findings provided a better insight into the utilization of ginsenoside Rg3 as a feasible approach to the treatment of arthritis characterized by upregulation of TNF-α.

## 2. Materials and Methods

### 2.1. Cell Culture and Reagents

TC28a2 Human Chondrocyte Cells were bought from Millipore Sigma (St. Louis, MO, USA). Cells were cultured in DMEM/F-12 medium supplemented with 10% fetal bovine serum (FBS) and penicillin (50 IU/mL)/streptomycin (50 μg/mL). A 0.25% (*w*/*v*) Trypsin-0.53 mM ethylenediaminetetraacetic acid (EDTA) solution was used to passage cells. Cells were cultured in humidified air with 5% CO_2_ at 37 °C. FBS and EDTA were purchased from Thermo Fisher Scientific (Waltham, MA, USA). Ginsenoside Rg3, STR1720, SB203580, Ammonium pyrrolidinedithiocarbamate (PDTC), and MitoTEMPO were obtained from Millipore Sigma (St. Louis, MO, USA). The MitoSOX™ Red Mitochondrial Superoxide Indicator and JC-1 Dye were obtained from Thermo Fisher Scientific (Waltham, MA, USA). Anti-SIRT1, anti-SIRT3, anti-Acetylated-Lysine, anti- PGC-1α, anti-COX IV, anti-Bax, anti-cytochrome c, anti-Bcl-2, anti-β-actin, anti-p-p38, anti-p38, anti-Lamin-B1and anti-NF-κBp65 were bought from Cell Signaling Technology (Danvers, MA, USA). Anti-cyclophilin D (CypD) was bought from Thermo Fisher Scientific (Waltham, MA, USA). Secondary antibodies were obtained from Santa Cruz Biotechnology, (Dallas, TX, USA).

### 2.2. Immunoblotting

The total proteins were lysed by RIPA buffer. The proteins in SDS/PAGE were transferred to the PVDF membrane. After blocking with 5% BSA in TBST for 1 h, the membrane was washed by TBST and incubated with primary antibodies for 18 h at 4 °C. After incubation of primary antibodies, PVDF membranes were incubated with HRP-conjugated secondary antibody for 1 h at 37 °C. Membranes were washed three times before the next step. PVDF membranes were detected with the enhanced chemiluminescence (ECL) system.

### 2.3. Investigation of Mitochondrial Biogenesis and Mitochondrial Membrane Potential

The Real-time PCR assay was used to study mitochondrial DNA (mtDNA) numbers. The primers of mitochondrial complex II: Sense primer 5′-CAAACCTACGCCAAAATCCA-3′ and antisense primer 5′-GAAATGAATGAGCCTACAGA-3′ and β-actin: Sense primer 5′-AGGTCATCACTATTGGCAACGA-3′ and antisense primer 5′-CACTTCATGATGGAATTGAATGTAGTT-3′. PCR was assayed by SYBR Green on an ABI 7000 sequence detection system according to the protocol. N-nonyl acridine orange (NAO) was selected for examining the mitochondrial mass. Cells were incubated with 5 µM NAO for 30 min at 37 °C, and cells were assayed by flow cytometry. The JC-1 was bought to investigate mitochondrial membrane potential. After TNF-α treatment, cells were rinsed with medium and then loaded with 5 μM JC-1 for 15 min at 37 °C. Cells were investigated by flow cytometry.

### 2.4. Mitochondrial Superoxide Indicator

At the end of TNF-a treatment. Wells were washed two times with PBS. 5 μM MitoSOX™ reagent was loaded to wells for 10 min incubation 37 °C. The trypsin/EDTA was used for cell detachment. Cells were assayed by flow cytometry.

### 2.5. Preparation of Nuclear/Cytosolic Extracts and Mitochondria Isolation

Nuclear and cytosolic extracts were isolated with a NE-PER™ Nuclear and Cytoplasmic Extraction kit (Catalog number: 78833, Thermo Fisher Scientific, Waltham, MA, USA). The nuclear extracts (supernatants) were stored at −80 °C until use. The NF-kB p65 Transcription Factor Assay Kit (Catalog number: ab133112, Abcam, Cambridge, UK) was used for testing the activity of NF-kB. The Mitochondria fraction was obtained by a Mitochondria Isolation Kit (Catalog number: 89874, Thermo Fisher Scientific, Waltham, MA, USA).

### 2.6. Co-Immunoprecipitation (Co-IP)

To examine the protein–protein interaction, Co-IP and Western blots were used as follows. TC28a2 human chondrocyte cells were washed three times with PBS, then whole-cell extracts were prepared via lysing cells in a lysis buffer. The primary antibody was added to the supernatant and incubated with a shaker for 20 min at 37 °C. After adding a protein A-agarose bead suspension, the mixture was further incubated with rotation for 2 h at 4 °C. The precipitates were washed three times using pre-cold lysis buffer. Then the beads were resuspended in a 1× sample. The immunoprecipitates or the whole-cell lysates (Input) were resolved by SDS-PAGE and transferred to PVDF membranes and then followed the protocol of Western blotting assay.

### 2.7. Investigation of Apoptosis

Apoptotic cells were analyzed by the ApopTag^®^ Peroxidase In Situ Apoptosis Detection Kit (Calbiochem). After TNF-α treatment, chondrocytes were rinsed three times in PBS before fixation for 30 min at 37 °C by 4% paraformaldehyde. Next, cells were washed in PBS before incubation in the prepared solution (0.1% Triton X-100, 0.1% sodium citrate) for 5 min. Cells were then incubated with 1xTUNEL reaction mixture in a humidified atmosphere for 60 min at 37 °C in the dark, washed two times in PBS, and assayed by flow cytometry.

### 2.8. Transfection with Small-Interfering RNA

ON-TARGET plus SMART pool small-interfering RNAs (siRNAs) for si-Controls were obtained from Dharmacon Research (Lafayette, CO, USA). si-SIRT1, si-PGC-1a, and si-SIRT3 were purchased from Santa Cruz. Transient transfection was performed using INTERFERin siRNA transfection reagent (jetPRIME^®^, polyplus transfection, UK) according to the manufacturer’s guide.

### 2.9. IL-8 and MMP-9 Release

Cells were seeded in 24-well plates at 0.5 × 10^5^ cells. After 48 h, cells were treated TNF-α for 24 h. Cell supernatants were removed and assayed for IL-8 and MMP-9 concentrations using an ELISA kit obtained (Catalog number: D8000C and DY911, R & D Systems, Minneapolis, MN, USA).

### 2.10. Statistical Analyses

The results are expressed as mean ± SD. Statistical analyses were performed using a one-way or two-way ANOVA, followed by a Tukey’s test as appropriate. A *p*-value < 0.05 was considered statistically significant.

## 3. Result

### 3.1. Administration of Ginsenoside Rg3 Reverses the TNF-α-Inhibited SIRT1 Expression

**Figure 1:**
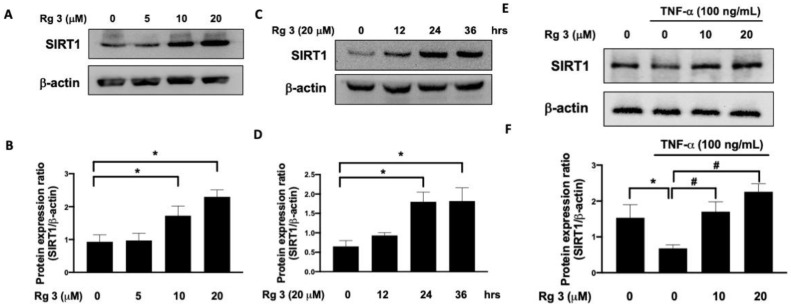
Rg3 enhances SIRT1 expression in chondrocytes. The expression levels of SIRT1 in C28a2 human chondrocytes treated with Rg3 were increased in dose-dependent (**A**,**B**) and time-dependent (**C**,**D**) manners. Representative Western blot images (**E**) and relative densitometric bar graphs of SIRT1/b-actin (**F**) in chondrocytes stimulated to 100 ng/mL TNF-a for 24 h were shown. (* indicating *p*  <  0.05 compared with the control group; # indicating *p*  <  0.05 compared to TNF-α-stimulated cells).

Sirtuin 1 (SIRT1) has been suggested to exhibit positive effects on cartilage under stress conditions [14], and ginsenoside Rg3 was reported to upregulate SIRT1 [15]. As shown in Figure 1A,B, administration of ginsenoside Rg3 dose-dependently elevated the expression of SIRT1 in TC28a2 human chondrocyte cells. The expression of SIRT1 was also increased following ginsenoside Rg3 treatment in a time-dependent manner (Figure 1C,D). Since SIRT1 exerted protective effects against the TNFα-mediated injury in OA chondrocytes [16], we examined if ginsenoside Rg3 could upregulate SIRT1 expression in response to TNFα stimulation. We showed that TNFα inhibited the expression of SIRT1 in TC28a2 human chondrocytes, whereas administration of ginsenoside Rg3 increased the level of SIRT1 in a dose-dependent fashion (Figure 1E,F).

### 3.2. Prevention of TNF-α-Induced Acetylation of CypD Using Ginsenoside Rg3 Is Mediated by SIRT1/PGC-1α/SIRT3 Pathway

**Figure 2:**
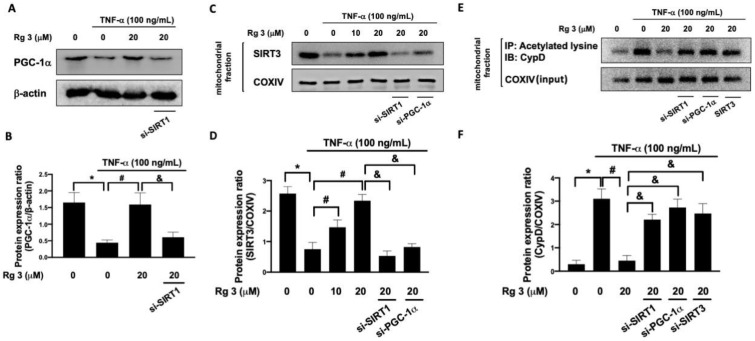
Rg3 promotes PGC-1α expression through modulation of SIRT1. Representative Western blot images (**A**) and relative densitometric bar graphs of PGC-1α (**B**) in chondrocytes stimulated to 100 ng/mL TNF-a for 24 h were shown. In some cases, cells were transfected with siRNAs 48 h before treatment with Rg3. Mitochondria SIRT3 expression levels were investigated by Western blotting assay (**C**,**D**). Representative Western blot images (**E**) and quantification of expression (**F**) of acetylated cyclophin D (CypD) and COXIV in the mitochondrial fraction. (* indicating *p*  <  0.05 compared with the control group; # indicating *p*  <  0.05 compared to TNF-α-stimulated cells; & indicating *p*  <  0.05 compared to TNF-α plus Rg3 cells).

It has been known that SIRT1 directly interacts with the peroxisome proliferator-activated receptor-gamma coactivator 1-alpha (PGC-1α) [17] and their interaction plays an integral role in energy metabolism. Additionally, sirtuin 3 (SIRT3) is the main sirtuin involved in acetylation/deacetylation of mitochondrial proteins and a downstream target of PGC-1α, which participates in the regulation of mitochondrial biogenesis [18]. We demonstrated that the TNFα-inhibited expression of PGC-1α was rescued by ginsenoside Rg3 treatment, and this effect was abolished when SIRT1 was knockdown (Figure 2A,B). Likewise, we showed that the downregulation of SIRT3 by TNFα was prevented in ginsenoside Rg3-treated cells but this result was not seen in cells transfected with si-SIRT1 or si-PGC-1α (Figure 2C,D).

Cyclophilin D (CyPD) has been revealed to modulate mitochondrial membrane permeability and oxidative damage-induced cell death [19]. Also, the SIRT3-mediated deacetylation of CypD has been shown to regulate the mitochondrial permeability transition pore (mPTP) [20]. As evident in Figure 2E,F, the results of immunoprecipitation demonstrated that TNFα increased the acetylated CyP-D level in mitochondria which was attenuated by treatment with ginsenoside Rg3. However, this phenomenon was blocked in cells transfected with si-SIRT1, si-PGC-1α, or si-SIRT3 (Figure 2E,F). Thus, our results demonstrated that the suppressive effect of ginsenoside Rg3 on the acetylation of CypD by TNF-α was mediated through SIRT1/PGC-1α/SIRT3 signaling.

### 3.3. Ginsenoside Rg3 Ameliorates the TNF-α-Induced Mitochondrial Dysfunction and Apoptosis via SIRT1/PGC-1α/SIRT3 Pathway

**Figure 3:**
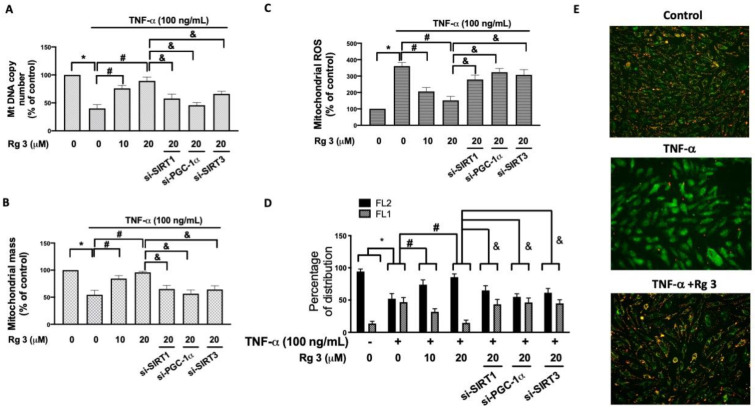
Rg3 up-regulates mitochondrial biogenesis, reduces ROS, and maintains mitochondrial function under TNF-α stimulation. The mitochondrial DNA copy number (**A**) and mitochondrial mass (**B**) were examined after administration of TNF-α for 24 h. In some cases, cells were transfected with siRNAs 48 h before treatment with Rg3. Dihydroethidium (DHE) was used for the investigation of mitochondrial ROS (**C**). Percentage of cells expressing JC-1 aggregates (red fluorescence; FL2) and JC-1 monomers (green fluorescence; FL1) were assessed using flow cytometry (**D**) or a fluorescence microscope (**E**). (* indicating *p*  <  0.05 compared with the control group; # indicating *p*  <  0.05 compared to TNF-α-stimulated cells; & indicating *p*  <  0.05 compared to TNF-α plus Rg3 cells).

In human knee OA chondrocytes, it has been found that the mitochondrial biogenesis capacity was reduced with loss of mitochondrial DNA (mtDNA) content and mass as well as the suppressed mitochondrial function [21]. Wang et al. showed that the decreased mitochondrial biogenesis was associated with the reduced decreased expression of SIRT-1 and PGC-1α [21]. In agreement with these findings, we showed TNF-α downregulated the mtDNA DNA copy and mitochondrial mass, which was dose-dependently reverted by ginsenoside Rg3 treatment (Figure 3A,B). Moreover, our results suggested that these effects were mediated by SIRT1/PGC-1α/SIRT3 signaling as silencing of SIRT1, PGC-1α or SIRT3 prevented this change (Figure 3A,B).

Next, we examined mitochondrial ROS production and showed ginsenoside Rg3 was able to mitigate the TNF-α-induced ROS, whereas inhibition of SIRT1, PGC-1α, or SIRT3 eliminated this effect (Figure 3C). Given that the opening of mPTP resulted in the collapse of the mitochondrial membrane potential and subsequent release of cytochrome *c* [22], we then assessed the mitochondrial membrane potential using JC-1 dye. The JC-1 dye can enter the mitochondria of healthy cells and form J aggregates, which exhibit excitation and emission in the red spectrum (FL2) instead of green (FL1; JC-1 monomer) that are seen in the unhealthy or apoptotic cells. As expected, cells treated with TNF-α displayed a reduced percentage of JC-1 red fluorescence (FL2) and increased green fluorescence (FL1).Rg3 treatment protected against TNF-α-impaired mitochondrial membrane potential, while inhibition of SIRT1/PGC-1α/SIRT3 pathway prevented this phenomenon (Figure 3D). Results from immunofluorescence staining showed similar findings that the majority of the TNF-α-stimulated cells exhibited green fluorescence, which was avoided with the administration of ginsenoside Rg3 (Figure 3E).

**Figure 4:**
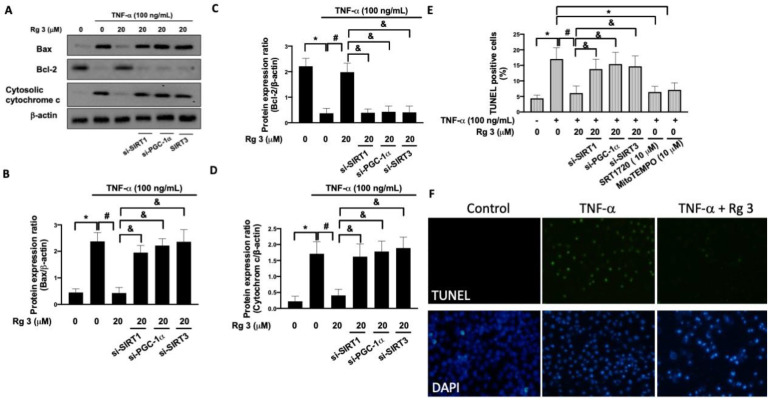
Rg3 protects against TNF-α-caused apoptosis in human chondrocytes through SIRT1/PGC-1/SIRT3 pathway. Representative Western blot images (**A**) and relative densitometric bar graphs of Bax (**B**), Bcl-2 (**C**), and cytochrome c (**D**) in chondrocytes stimulated to 100 ng/mL TNF-α for 24 h were shown. In some cases, cells were transfected with siRNAs 48 h before treatment with Rg3. TUNEL assay was used for the investigation of apoptotic cells using flow cytometry (**E**) and the fluorescent microscope (**F**). The selective activator SIRT1, SRT1720, and the inhibitor of mitochondrial ROS, mitoTEMPO, were pre-treated 2 h before TNF-α-stimulation. (* indicating *p*  <  0.05 compared with the control group; # indicating *p*  <  0.05 compared to TNF-α-stimulated cells; & indicating *p*  <  0.05 compared to TNF-α plus Rg3 cells).

To ascertain the mitochondrial dysfunction and ROS production affected apoptosis, we measured the expression of Bax, Bcl-2, and cytochrome *c*. Results from Western blot showed that TNF-α upregulated the expression of Bax and cytochrome *c* with downregulation of Bcl-2, which were all abrogated after knockdown of SIRT1, PGC-1α, or SIRT3 (Figure 4A–D). Additionally, we observed that the TNF-α-increased percentage of TUNEL positive apoptotic cells was downregulated by ginsenoside Rg3 administration (Figure 4E). Aside from demonstrating that inhibition of SIRT1/PGC-1α/SIRT3 signaling blocked the effects from ginsenoside Rg3, we also showed that the increased percentage of TUNEL positive cells by TNF-α were abolished in the presence of SRT1720 (a selective SIRT1 synthetic activator) or MitoTEMPO (a specific scavenger of mitochondrial superoxide) (Figure 4E). These findings suggested that the ROS production resulting from the TNF-α-inhibited SIRT1/PGC-1α/SIRT3 signaling contributed to apoptosis. Taken together, these data demonstrated the implication of SIRT1/PGC-1α/SIRT3-mediated pathway in the attenuation of TNF-α-induced mitochondrial dysfunction, oxidative stress, and apoptosis by ginsenoside Rg3.

### 3.4. Ginsenoside Rg3 Suppresses the TNF-α-Stimulated p38 MAPK Phosphorylation and NF-κB Activation through SIRT1/PGC-1α/SIRT3 Signaling

**Figure 5:**
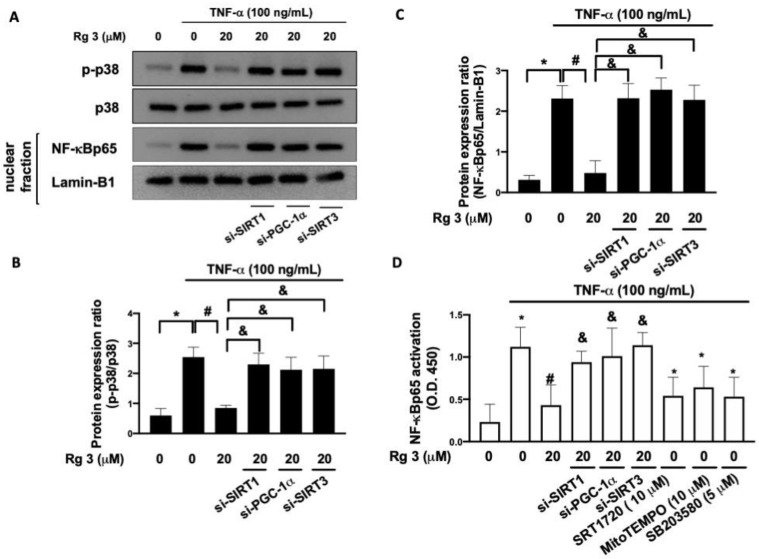
Rg3 mitigates TNF-α-caused NF-kB activation. Representative Western blot images (**A**) and relative densitometric bar graphs of p-p38/p38 (**B**) and NF-kBp65/Lamin-1 (**C**) in chondrocytes stimulated to 100 ng/mL TNF-a for 24 h were shown. In some cases, cells were transfected with siRNAs 48 h before treatment with Rg3. NF-kB activation was also confirmed by the NF-αB activity kit (**D**). In some cases, cells were transfected with siRNAs 48 hrs before treatment with Rg3. The selective activator SIRT1, SRT1720, and the inhibitor of mitochondrial ROS, mitoTEMPO, and the inhibitor of MAPK p38, S203580, were pre-treated 2 h before TNF-α stimulation. (* indicating *p*  <  0.05 compared with the control group; # indicating *p*  <  0.05 compared to TNF-α-stimulated cells; & indicating *p*  <  0.05 compared to TNF-α plus Rg3 cells).

Activation of p38 mitogen-activated protein kinase (MAPK) is crucial in TNF-α-related arthritis [23], and the p38 MAPK/NF-kB axis has been shown to mediate the inflammatory response in chondrocytes [24]. We showed that administration of TNF-α upregulated the expression of phosphor-p38 and NF-kBp65, whereas suppression of SIRT1/PGC-1α/SIRT3 signaling inhibited the increased phosphorylation of p38 MAPK and expression of NF-kBp65 (Figure 5A–C). Analysis of NF-kBp65 activation exhibited a similar finding (Figure 5D). Moreover, we showed that the TNF-α-induced NF-kBp65 activation was diminished when SRT1720, MitoTEMPO, or SB203580 (a p38 MAPK inhibitor) was employed (Figure 5D), suggesting that the TNF-α-induced accumulation of ROS participated in the activation of p38 /NF-kB signaling. Collectively, our findings implied that TNF-α-associated p38 MAPK/NF-kB activation was mediated by the SIRT1/PGC-1α/SIRT3 pathway, which could be lessened by ginsenoside Rg3 treatment.

### 3.5. Ginsenoside Rg3 Inhibits the TNF-α-Increased Production of IL-8 and MMP-9 via SIRT1/PGC-1α/SIRT3/p38 MAPK/NF-κB Pathway

**Figure 6:**
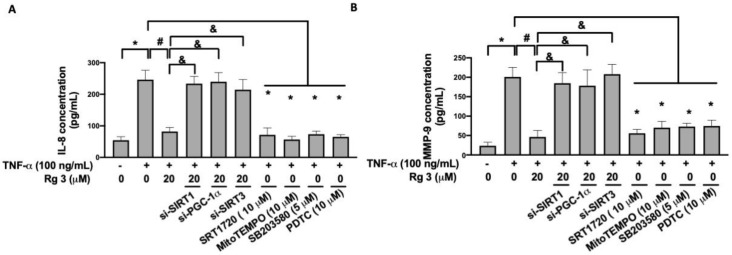
Rg3 reduces TNF-α-caused pro-inflammatory response and MMP-9 activation in human chondrocytes through SIRT1/PGC-1/SIRT3/NF-kB pathway. Chondrocytes were stimulated to 100 ng/mL TNF-α for 24 h were shown. The selective activator SIRT1, SRT1720, and the inhibitor of mitochondrial ROS, mitoTEMPO, the inhibitor of MAPK p38, S203580, and the inhibitor of NF-αB, PDTC, were pre-treated 2 h before TNF-α stimulation. The culture medium was collected for IL-8 (**A**) and MMP-9 (**B**) ELISA assay. (* indicating *p*  <  0.05 compared with the control group; # indicating *p*  <  0.05 compared to TNF-α-stimulated cells; & indicating *p*  <  0.05 compared to TNF-α plus Rg3 cells).

Translocation of NF-κB has been known to be related to the upregulation of IL-8 [25] and MMP-9 [26] in chondrocytes. We demonstrated that ginsenoside Rg3 abolished the TNF-α-elicited IL-8 or MMP-9 production, but this inhibitory effect was reversed by downregulation of SIRT1/PGC-1α/SIRT3 signaling (Figure 6A,B). Besides, activation of SIRT1 by SRT1720, suppression of mitochondrial ROS by MitoTEMPO, inhibition of p38 MAPK by SB203580, or blockade of NF-κB signaling by pyrrolidine dithiocarbamate (PDTC) all reduced the TNF-α-induced IL-8 or MMP-9 production (Figure 6A,B). Altogether, these results indicated that the elevation of IL-8 or MMP-9 by the p38 MAPK/NF-kB axis was due to the accumulation of SIRT1/PGC-1α/SIRT3-mediated oxidative stress. Besides, administration of ginsenoside Rg3 may be beneficial to alleviate the TNF-α-induced chondrocyte injury by reduction of IL-8 or MMP-9.

## 4. Discussion

Over the past few years, multiple studies have demonstrated the anti-inflammatory effect of ginsenoside Rg3 on various diseases. For example, it has been shown to ameliorate hepatic injury by suppressing PI3K/AKT pathway-mediated inflammation [27]. Ginsenoside Rg3 also induced the polarization of the M2 macrophages to accelerate the resolution of inflammation, and it inhibited the mast cell-mediated allergic inflammation via MAPK/NF-κB signaling [28]. In the IL-1β-stimulated inflamed A549 cells (adenocarcinoma human alveolar basal epithelial cells) and human asthmatic airway epithelial tissues, ginsenoside Rg3 was shown to downregulate the NF-κB activity and cyclooxygenase-2 (COX-2) as well as other NF-κB-mediated cytokines, such as IL-4 and TNF-α [29]. Besides, a couple of studies suggested that ginsenoside Rg3 exerted cardioprotective effects by alleviating inflammation via the SIRT1/NF-κB pathway [30,31]. In agreement with these findings, we showed that administration of ginsenoside Rg3 exhibited anti-inflammatory properties to protect chondrocytes via upregulation of SIRT1.

SIRT1 is a class III histone deacetylase and is involved in numerous biological processes, such as stress responses, DNA damage, and inflammation. The expression of the SIRT 1 was detected in normal and diseased chondrocytes as well as synovial tissues [32,33]. It was found that the expression level of SIRT1 in chondrocytes from OA patients was reduced [32], whereas its expression in RA synovial tissues was elevated [33]. Several studies have demonstrated that SIRT1 expression and activity were decreased after treatment with catabolic stimuli IL-1β [32] or TNF-α [34] in chondrocytes, which was consistent with our findings. Moreover, the expression of SIRT1 in articular cartilage was found to be negatively associated with the severity of knee OA [35]. It has been demonstrated that silencing of SIRT1 induced the OA-like gene expression alteration, such as upregulation of collagen type X alpha 1 chain (COL10A1) and ADAMTS-5 (an extracellular matrix-degrading enzyme), suppression of chondrogenic markers SOX9, collagen type 2 alpha 1 chain (COL2A1) and aggrecan [36,37]. SIRT1 has been proven to enhance chondrocyte survival by inhibition of protein tyrosine phosphatase 1B to reduce apoptosis [38] and directly activate autophagy [37], a homeostatic mechanism in normal cartilage [39]. In addition, SIRT1 mitigated the nitric oxide (NO)-induced apoptosis [32] and regulated the TNF-α-induced inflammation in human chondrocytes [40]. It also downregulated the IL-1β-increased MMP-13 and ADAMTS-5 as well as diminished the acetylation of NF-κB p65 [41]. In line with these findings, we demonstrated that upregulation of SIRT1 by ginsenoside Rg3 abrogated the TNF-α-increased apoptosis. Also, it downregulated the TNF-α-induced production of IL-8 and MMP-9 via SIRT1/PGC-1α/SIRT3/p38 MAPK/NF-κB pathway.

The endogenous SIRT1 has been shown to interact with p65 and deacetylate it in human chondrocytes [41], and we demonstrated that TNF-α-elicited oxidative stress further enhanced the activation of NF-κBp65 via upregulation of p38 MAPK. It is well-known that once NF-κB is translocated into the nucleus, it stimulates the transcription of various genes regarding inflammation, apoptosis, cell proliferation, and cell cycle. In chondrocytes, inhibition of NF-κBp65 by siRNA has been known to reduce the IL-1β or TNF-α-upregulated COX-2, nitric oxide synthase-2 (NOS-2), and MMP-9 [42]. Furthermore, our previous work has shown that TNF-α-induced NF-κB activation led to an exaggerated inflammatory response with elevated levels of COX-2 and IL-8 [43]. Also, TNF-α-elicited p38 MAPK mediated the aberrant mitochondrial biogenesis and increased oxidative stress, which resulted in apoptosis in chondrocytes [44]. In the present study, we showed that activation of the p38 MAPK/NF-κBp65 axis resulted in upregulation of IL-8 and MMP-9, which may contribute to cartilage damage. Besides, our data suggested that TNF-α triggered p38 MAPK/NF-κBp65 axis via the accumulation of oxidative stress through SIRT1/PGC-1α/SIRT3 pathway.

SIRT3 is a mitochondrial nicotinamide adenine dinucleotide (NAD^+^)-dependent protein deacetylase that belongs to the silent information regulator 2 (SIR2) family [45]. It has been revealed that the expression of SIRT3 was decreased in OA chondrocytes [46,47], which compromised mitochondrial function [46]. Acetylation of mitochondria proteins typically decreased mitochondrial integrity and function [48]. Multiple lines of evidence suggested that the acetylation status in mitochondria was under the regulation of SIRT3 [49,50,51]. SIRT3 has been shown to enhance the activity of antioxidant superoxide dismutase 2 (SOD2) by deacetylating forkhead box class O 3a (FOXO3a) [52]. SIRT3 is also implicated in the mitochondrial redox status via the deacetylation of isocitrate dehydrogenase 2 (IDH2) [53]. In this study, we showed that SIRT3 modulated the deacetylation of CypD to control the opening of mPTP in chondrocytes, which was in accordance with other studies showing that SIRT3 mediated the deacetylation of CypD in aging-related cardiac hypertrophy [20] or breast carcinoma [54]. Frequent opening of mPTP can lead to mitochondrial dysfunction, and we did observe the impaired mitochondrial biogenesis, elevation of ROS, and apoptosis. In addition to maintaining mitochondrial homeostasis, SIRT3 also participated in the TNF-α-inhibited aggrecan and collagen II, and TNF-α-induced MMP-13 and ADAMTS-5 in chondrocytes [47]. Our results conformed with these findings showing that SIRT3 played a role in the TNF-α-elicited MMPs production.

Numerous studies have shown that PGC-1α elevated the expression of SIRT3, which was crucial to the maintenance of the mitochondrial biogenesis [18,55]. It has been revealed that PGC-1α activated SIRT3 via co-activation of the orphan nuclear receptor Err (estrogen-related receptor)-α, which could bind to the promoter region of SIRT3 in brown adipocytes or HepG2 cells [18,55]. On the other hand, PGC-1α activity is modulated by the network between phosphorylation through AMP-activated protein kinase (AMPK) and deacetylation via SIRT1 [56]. In chondrocytes, it has been demonstrated that AMPK regulated PGC-1α expression via SIRT1 [21]. Besides, the decreased expression of mitochondrial respiratory complexes I to IV and ATP synthase (complex V) along with lower oxygen consumption and intracellular ATP level was observed, indicating the impaired mitochondrial biogenesis in OA chondrocytes [21]. Our data was associated with these findings regarding the TNF-α-induced mitochondrial dysfunction in chondrocytes was mediated by SIRT1/PGC-1α axis.

This present study has several limitations, for example, we did not confirm the therapeutic effects of Rg3 in the animal model. Second, we used SV40-transformed chondrocytes for in vitro assay, which might be more resistant to stress and stimulation compared to primary human chondrocytes. In the future, we will use primary human chondrocytes to further confirm our hypothesis.

**Figure 7:**
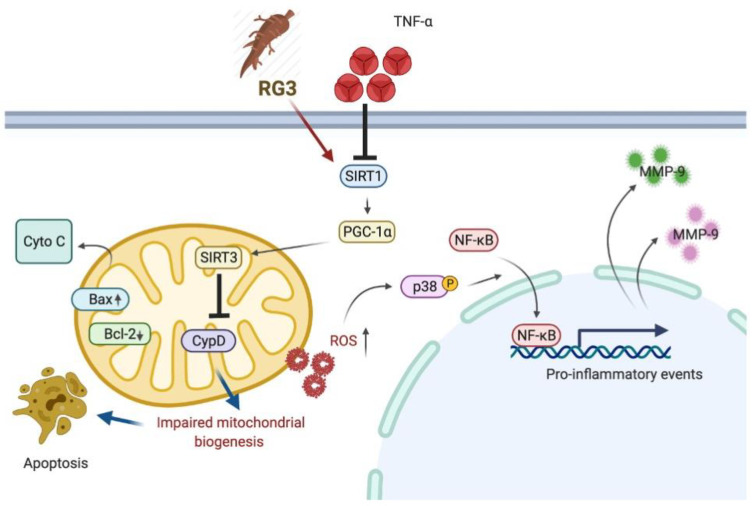
Proposed mechanisms of Rg3 in TNF-α-caused chondrocytes dysfunction. Rg3 activates SIRT1/PGC-1α/SIRT3 pathway, thereby inhibiting the TNF-α-elicited acetylation of CypD. Rg3 improves mitochondrial biogenesis, downregulates ROS, and apoptosis. Rg3 diminished the oxidative stress-associated p38 MAPK/NFκBp65 axis through upregulation of SIRT1/PGC-1α/SIRT3 signaling, leading to amelioration of IL-8 and MMP-9 secretion in chondrocytes with TNF-α stimulation.

In conclusion, we showed that ginsenoside Rg3 possesses the protective effects against the TNF-α-induced cartilage damage via upregulation of SIRT1. Our results demonstrated the activation of SIRT1/PGC-1α/SIRT3 pathway by ginsenoside Rg3 downregulated the TNF-α-elicited acetylation of CypD, leading to the improved mitochondrial biogenesis, downregulation of ROS, and the subsequent apoptosis. Moreover, we proved that administration of ginsenoside Rg3 diminished the ROS-associated p38 MAPK/NF-κBp65 axis through upregulation of SIRT1/PGC-1α/SIRT3 signaling, leading to amelioration of IL-8 and MMP-9 production in chondrocytes with TNF-α stimulation (Figure 7). Our findings suggested that ginsenoside Rg3 may be used as a supplement to mitigate inflammation and prevent cartilage degradation in arthritis patients.

## Data Availability

The data presented in this study are available in the article.
